# Geographical Features and Seroprevalence of *Borrelia burgdorferi* in Erzincan, Turkey

**Published:** 2018-12-25

**Authors:** Aytekin Cikman, Merve Aydin, Baris Gulhan, Faruk Karakecili, Levent Demirtas, Ozan Arif Kesik

**Affiliations:** 1 Department of Medical Microbiology, School of Medicine, Erzincan University, Erzincan, Turkey; 2 Department of Medical Microbiology, School of Medicine, KTO Karatay University, Konya, Turkey; 3 Department of Infectious Diseases, School of Medicine, Erzincan University, Erzincan, Turkey; 4 Department of Internal Medicine, School of Medicine, Erzincan University, Erzincan, Turkey; 5 Department of Geography, Faculty of Arts and Sciences, Erzincan University, Erzincan, Turkey

**Keywords:** *Borrelia burgdorferi*, Seroprevalence, Geographical features, Altitude, Slope degree, Turkey

## Abstract

**Background:**

We aimed to determine the geographical features and seroprevalence of *Borrelia burgdorferi* in Erzincan, Turkey, which has a high tick population due to its geographical position and climatic conditions.

**Methods:**

From January to December 2014, 368 people living in Erzincan, northeastern Turkey were enrolled. *B. burgdorferi* IgG antibodies were investigated in the collected serum samples using the ELISA method in 2015. Positive and borderline results were confirmed using the Western Blot (WB) method.

**Results:**

*Borrelia burgdorferi* IgG positivity was found to be 4.1% by ELISA and 2.17% by WB. Of the seropositive people according to WB, 25% resided in areas within 2000m of rivers, 50% in areas with a slope of 0–5°, and 62.5% in areas with an altitude of lower than 1500 meters.

**Conclusion:**

The seroprevalence of Lyme borreliosis was high in Erzincan, particularly among people engaged in animal husbandry in rural areas. In addition, the seroprevalence of *Borrelia* varied according to geographical features, increasing in areas with a lower slope and altitude.

## Introduction

Lyme borreliosis (LB), also known as Lyme disease, is the most common tick-borne infectious disease caused by spirochetes of the *Borrelia burgdorferi* sensu lato (s.l.) complex and is transmitted by *Ixodes* ticks (1, 2). To date, 19 different species have been identified in the *B. burgdorferi* s.l. complex. LB is mainly caused by three pathogenic genomic species: *Borrelia burgdorferi* sensu stricto (*B. burgdorferi*), *B. garinii*, and *B. afzelii* (3, 4).

There are 14 different species of ticks in the ‘*Ixodes ricinus* complex’, in which *Borrelia* continues its life cycle in natüre (5). In addition to the *Ixodes*, which are the vector of spirochetes, other hematophagous insects, rodents and some warm-blooded animals such as birds are also important in the ecological transmission of the disease (6). LB seroprevalence is increased in areas where these ticks live and cases of tick bites are commonly seen. LB is known to have a widespread distribution, particularly in forests and woodlands (7, 8). The risk areas for ticks in cities are green areas and parks. Therefore, the highest risk group for LB is forest and agricultural workers, hunters, livestock farmers, and individuals living in areas with a large tick population (9, 10).

LB has different clinical episodes. A typical lesion in the early stage of the disease is erythema chronicum migrans. However, the majority of patients also present with fever, flulike symptoms, and regional lymphadenomegaly (9, 11–13). In untreated cases, systemic manifestations such as musculoskeletal involvement, cardiovascular, and neurologic involvement may also develop during the later stages of the disease due to hematogenous dissemination (11, 14).

It is difficult to diagnose LB due to the different clinical manifestation of overlapping symptoms and the high antigenic variability of *B. burgdorferi*. A two-step standard diagnostic protocol is recommended for the laboratory confirmation of LB. In the first step, an ELISA or an indirect fluorescent antibody (IFA) method is used for the detection of antibodies. In the second step, Western blot (WB) test is recommended to confirm the positive results obtained from the first step (15–17).

LB is the most common tick-borne infectious disease in the northern hemisphere, and climatic, economic and social changes in recent years have been reported to increase the incidence of the disease (6, 18, 19). Located in the northern hemisphere, Turkey has similar geographical and climatic conditions to many European countries, in which LB is frequently reported (20). *Ixodes* are the most common type of ticks observed in Turkey (21). However, in Turkey, there are only limited studies on the seroprevalence of LB.

Therefore, we aimed to determine the seroprevalence of LB in Erzincan located in the northeast of Turkey using the ELISA and WB methods and to evaluate some of the risk factors.

## Materials and Methods

### Study Area

The study was carried out from January to December 2014 in Erzincan, located between 39° 02′–40° 05′ north latitude and 38° 16′–40° 45′ east longitude in northeast Turkey. Erzincan comprises nine districts, Refahiye, Kemah, Kemaliye, Tercan, Çayırlı, İliç, Otlukbeli, Üzümlü, and Central district. Erzincan is located in the Kelkit Valley which has a large tick fauna. It has many rivers, streams, and wetlands, which facilitate farming and animal husbandry practices. Erzincan has the characteristics of a terrestrial climate characterized by relatively cold and rainy winters and hot and dry summers. The annual average temperature is 10.9 °C and the coldest and hottest months are January and July with average temperatures of −6.7 °C and 31.4 °C, respectively. Erzincan has an average rainfall of 380.6mm with the maximum being 633.1mm and the minimum being 206.1mm. The annual average humidity is 64.26% (22).

### Collection of blood samples

This study was conducted with the approval of Erzincan University Ethics Committee (Approval no: 2014/7).

The study was planned as cross-sectional epidemiological research. The sample size was determined using a cluster sampling method. Overall, 368 healthy volunteers were included in the study.

All the participants were informed about the study and signed the informed consent forms. The participants were also asked to complete a short questionnaire to reveal information on their gender, age, occupation, place of residence, engagement in animal husbandry, and tick exposure.

A 10mL venous blood sample was taken from all participants. The samples were transferred to the laboratory maintaining the cold chain and then centrifuged at 1610g for 10min to separate the sera and stored at −80 °C until the time of serological tests.

### Detection of antibodies using ELISA

*Borrelia burgdorferi* immunoglobulin G (IgG) antibodies were determined in all serum samples using a SERION ELISA classic kit (Institut Virion\Serion GmbH, Wurzburg, Germany) according to the manufacturer’s recommendations. The samples were diluted 1:100 using a dilution buffer. The standards and diluted samples were transferred to microtiter wells and incubated at 37 °C for 60min in a moist chamber. The residual serum was removed from the wells by washing four times with a wash buffer. Anti-human IgG conjugated to alkaline phosphatase was added and incubated at 37 °C for 30min in a moist chamber. The wells were washed four times with the wash buffer; then, substrate p-nitrophenyl phosphate was added and incubated at 37 °C for 30min, followed by the addition of a stop solution. The optic density was determined at 405nm (at the 650nm reference wavelength) using an Epoch ELISA spectrophotometer (BioTek Instruments, Inc., Winooski, VT, USA). Each kit was used with a negative control, positive control, and standards in duplicate. Interpretation of the results was performed assisted by Serion Easy Base 4PL software, and the results were expressed in units /milliliter (IgG, <3U/ml [negative], 3–5U/ml [borderline], >5 U/ml [positive]).

### Detection of antibodies using WB

The serum samples that had a positive or borderline value in the ELISA IgG test were further analyzed using a Viro-Blot kit (Viro-Immun Labor-Diagnostika GmbH, Germany) to confirm the presence of *B. burgdorferi* IgG antibodies according to manufacturer’s recommendations. Briefly, nitrocellulose strips containing electrophoresis-separated *B. afzelii*/*B. garinii*/ sensu stricto proteins were blocked and then incubated with 1020μl of the diluted serum sample (1:51) for 60min. The membrane strips were washed and incubated with alkaline phosphatase (AP)-conjugated anti-human IgG antibody for 30min. Following a final wash, strips were incubated with a chromogen-substrate solution for 10–15min, washed and air-dried on a rocking platform. The quantitative analysis of bands on each blot was carried out using the BLOTrix software (Viro-Immun Labor-Diagnostika GmbH, Germany). The software corrected the background and determined the cutoff values for positivity for the recombinant VlsE borrelial protein bands. The IgG assay was considered to be positive if five or more of the following ten bands were present: p17/p19 complex, p20/p21 complex, p25 (OspC), p30, p31 (OspA), p35, p45, p59/ p62 complex, p100, and VIsE.

### Mapping

ArcGIS 10.1 and Google Earth programs were used to draw the maps and conduct analyses. First, a map of Erzincan including its districts and river and stream branches was drawn to be used in the spatial analysis. Next, baseline data were created using the ArcGIS Basemap OpenStreetMap service. Then, the locations of the seropositive cases were detected in Google Earth program and transferred to ArcMap software. Addition, the relationship between seropositive cases and the altitude and slope degrees of their places of residence was evaluated. To determine the geographic correlations with seropositive samples, we obtained data from the Advanced Spaceborne Thermal Emission and Reflection Radiometer Global Digital Elevation Model (ASTER GDEM), National Aeronautics and Space Administration (NASA), and the Ministry of Economy, Trade, and Industry (METI). In addition, spatial analysis was performed through buffer analyses (Buffer, Multiple Ring Buffer) on the river and streams and their branches.

### Statistical analysis

The data were evaluated using the (IBM SPSS Statistics for Windows, Version 20.0, IBM Corp., Armonk, NY, USA, Released 2011). The values of the variables were expressed in mean±standard deviation and median (max–min) percent and frequency. The categorical data were analyzed by Fisher’s Exact Test and the Chi-square test. When the expected frequency was less than 20%, the Monte Carlo Simulation Method was used to evaluate and determine the frequencies to be included in the analysis. P< 0.05 were accepted as the significance levels for the tests.

## Results

Of the 368 individuals included in the study, 225 (61.1%) were female and the average age was calculated as 51.43±16.91 year. There was no statistically significant difference between the participants in terms of average age and gender (P= 0.578). Using the ELISA method, *B. burgdorferi* IgG antibodies were found to be positive in 15 individuals (4.1%) and the results of 36 individuals were within borderline value range. These 51 samples with positive or borderline values were further analyzed using the WB technique to determine their *B. burgdorferi* IgG levels. WB confirmed positivity in 8 of the 51 samples. Thus, the overall *B. burgdorferi* IgG positivity was calculated as 2.17% (8/368) (Table 1).

**Table 1.** The seroprevalence of *Borrelia burgdorferi* and associated risk factors

		**ELISA IgG (n=368)**			**WB (n=51)**		
	
		**Borderline (n=36)**	**Positive (n=15)**	**P**	**Borderline (n=17)**	**Positive (n=8)**	**P**
**Gender**	**Female**	22	11	0.405	10	5	0.861
	**Male**	14	4		7	3	
**Age**	≤**45**	10	6	0.391	4	3	0.468
	**> 45**	26	9		13	5	
**Animal farming**	**Yes**	28	12	0.010*	14	6	0.668
	**No**	8	3		3	2	
**Tick exposure**	**Yes**	16	3	0.010*	9	4	0.891
	**No**	20	12		8	4	
**Living area**	**Rural**	30	13	0.010*	15	7	0.010*
	**Urban**	6	2		2	1	

* P< 0.05

Among the 8 individuals with *B. Burgdorferi* positivity confirmed by the WB method, 7 lived in rural areas, 6 were engaged in animal farming and 4 were exposed to ticks. Three of these eight cases were men aged 65 to 75yr engaged in farming, lived in rural areas and were exposed to ticks. When all the individuals were evaluated, living in rural areas was found to be statistically significant in relation to ELISA and WB values (P= 0.010). Furthermore, tick exposure and engagement in animal farming had a statistically significant effect on the positivity of the ELISA IgG values, but not the positivity of the WB values (Table 1).

**Fig. 1.**
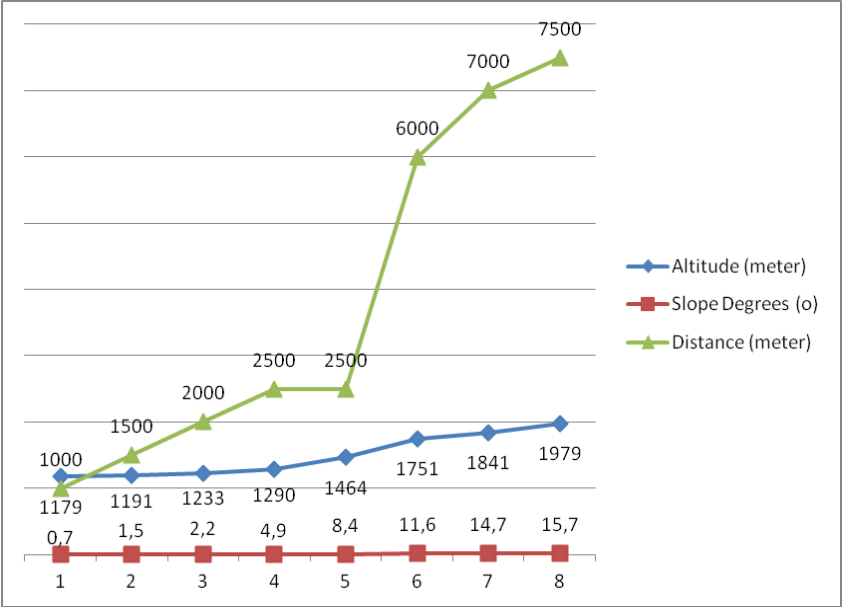
The relationship between IgG positivity and altitude (meter), slope degrees (°) and distance (meter) according to the WB method

**Fig. 2.**
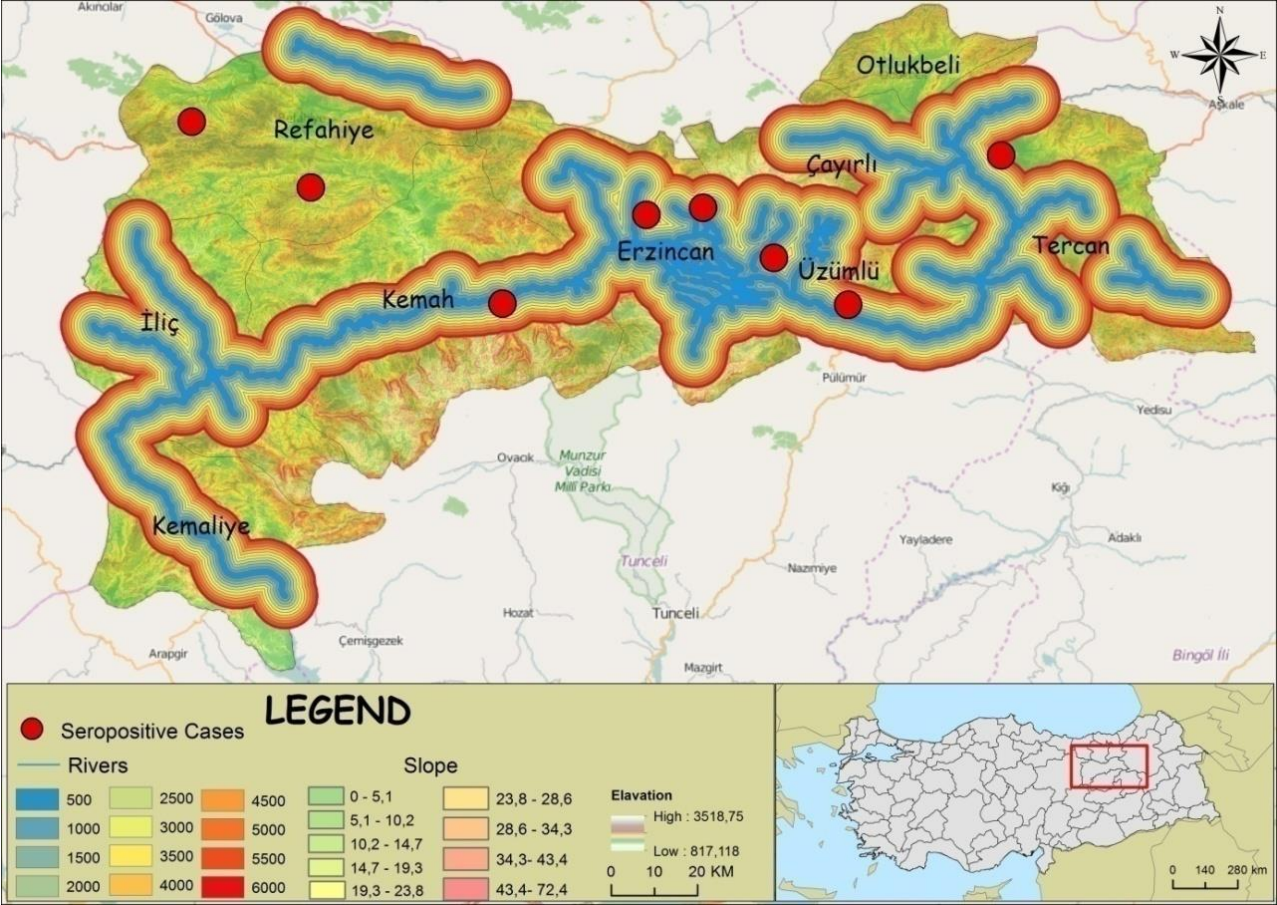
The geographical features of areas in which seropositive individuals lived according to the results of the WB method

The relationship between WB positivity and geographical features namely distance to rivers, altitude, and slope was investigated. Twenty-five percent of people with IgG positivity lived within 2000m of rivers or their main branches in the study area. The slope of the study area ranged from 0 to 72.44°. The slope degrees of the residential places of the seropositive people were 0–5° for 50%, 5–10° for 13%, and greater than 10° for 37%. Lastly, the study area had an altitude of 817 to 3518m above sea level, and 62.5% of seropositive cases lived in places with an altitude of fewer than 1500 meters (Fig. 1, 2).

## Discussion

LB poses serious problems among tick-borne diseases. LB is particularly well known in the northern hemisphere and is the most common infectious disease caused by ticks in North America and Europe (23, 24). The incidence of LB in Europe has been reported to range from 5% to 25% (25). The incidence of LB is high in the south of Scandinavia and the north of Mediterranean countries increasing from the west to the east. Central and Eastern Europe are the endemic areas in which the disease is most frequently seen (26).

Although different *Borrelia* species can cause LB, the most common agents in Europe are *B. afzelii* and *B. garinii* (27). Turkey has a similar tick fauna to many European countries (21). In this study, taking into consideration the reported results (21), we used an ELISA kit containing *B. burgdorferi* sensu stricto, *B. afzelii* and *B. garinii* antigens (second generation). In the second phase of the study, we employed a highly specific immunoblot method including the VlsE surface antigen, reported to have the highest sensitivity and specificity (28, 29).

In Turkey, although LB is not a notifiable infectious disease and it is relatively less known, studies conducted in recent years have revealed its prevalence. The seroprevalence of *B. burgdorferi* was evaluated among people engaged in agriculture and animal husbandry and reported it to be 3.3% (30). The *B. Burgdorferi* IgG antibody positivity in risk groups was found as 3.8% using the ELISA method (20). The WB technique confirmed the positive result in 0.9% of the cases. In the current study, we found a similar percentage of *B. burgdorferi* IgG positivity (4.1%) using ELISA compared to previous studies. However, the percentage of WB *B. burgdorferi* positivity (2.17%) was higher than previous reports. This higher ratio can be attributed to the current study area having a large tick fauna.

Agricultural workers, hunters, and livestock farmers form the risk group of LB. However, these groups also vary according to the natural habitat of different tick species (31, 32). In the current study, using the ELISA method, risk factors were determined to be living in a rural area, exposure to ticks, and engagement in animal husbandry. However, with the WB method, only living in rural areas was found to have a statistically significant effect on the incidence of LB. This can be explained by the lower number of positive cases detected by WB.

LB cases in Europe are generally reported between the latitudes of 40°N and 60°N. The incidence of LB also varies according to geographical location and species of ticks (9, 33). Tick species infected with *Borrelia* were more common in areas with an altitude lower than 1300 meters (9). In Norway, LB seroprevalence was lower in inland areas but increased in coastal areas and those close to the south. However, the seroprevalence of LB also differed according to geographical characteristics (34). Similarly, in the current study, 62.5 % of the seropositive individuals lived in areas with an altitude below 1500m. Furthermore, concerning the slope degrees of the places of residence of seropositive cases, 50% had a 0–5° slope. The results of the current study are similar to those reported for Norway in terms of the higher percentage of seropositive individuals living in coastal areas. LB is more frequently seen in areas with a lower slope and lower altitude. As another geographic feature, we also examined the distance of the positively identified people to the rivers in the study area and found that 25% lived within 2000 m from the rivers. Contrary to our expectation, this data indicates that LB is more common in areas away from wetlands.

The seroprevalence of LB will vary according to the geographical characteristics. This is one of the rare studies, in which the relationship between LB seroprevalence and geographical features (such as distance to river, altitude, and slope degree) was explored. In addition to geographical features, environmental factors such as climate, living conditions and the presence of reservoir animals affect LB seropositivity. The current study is also important in terms of being the first to investigate LB seroprevalence in Erzincan, an area with a large tick fauna.

## Conclusion

The seroprevalence of LB was found to be high in Erzincan, particularly among people engaged in animal husbandry and exposed to ticks. The seroprevalence of *Borrelia* varies according to geographical features and is higher in areas with a lower slope degree and altitude.
